# Susceptibility to long-term misinformation effect outside of the laboratory

**DOI:** 10.3402/ejpt.v4i0.19864

**Published:** 2013-05-02

**Authors:** Miriam J. J. Lommen, Iris M. Engelhard, Marcel A. van den Hout

**Affiliations:** Department of Clinical and Health Psychology, Utrecht University, Utrecht, The Netherlands

**Keywords:** Memory, individual differences, misinformation effect

## Abstract

**Objective:**

To test the effect of misinformation outside of the laboratory and to explore correlates of the effect, including arousal, cognitive ability, and neuroticism.

**Method:**

About 2 months before deployment to Afghanistan, 249 soldiers enrolled in this study, which was embedded in a larger project. Two months after deployment, participants were interviewed about stressors on deployment and they received subtle misinformation about a fictional event on deployment. Seven months later, they were retested, and completed a questionnaire about events on deployment.

**Results:**

At 9 months, a total of 26% of participants reported that they had experienced the fictional event, although 7 months earlier they said they had not experienced it. Logistic regression analyses revealed that lower cognitive ability and a combination of high arousal and more stressors on deployment were related to higher susceptibility to the misinformation effect.

**Conclusions:**

Results suggest that information provided by another source may be incorporated into related autobiographical memory, particularly for individuals with lower cognitive ability, high arousal at the time of encoding the information and more related experiences.

How often do you answer the question “How do you know?” with “Because I remember”? We rely on memory every day and may assume that it is veridical, especially when memories are vivid and detailed. However, memory is malleable, and this was clearly demonstrated by Loftus and Palmer's (1974) classic study about the effects of suggestive questions on eyewitness reports. In this study, participants were shown a film about a car accident. Then some of them were asked to estimate the speed of the cars when they “smashed” into each other, whereas others received the same question in which the word “smashed” had been replaced by “hit”. The “smashed” group made higher speed estimates than the “hit” group, and was more likely to report 1 week later that they had seen broken glass in the film, even though no broken glass had been shown. In another study by Loftus, Miller, and Burns (1978), participants were shown a series of pictures about an auto-pedestrian accident, including a picture with a red Datsun at a *stop* sign for half of the group, and with a *yield* sign for the other half of the group. Then both groups were asked “Did another car pass the red Datsun while it was stopped at the *yield* sign?”. In a subsequent memory task that involved a forced choice between the picture of the *yield* sign and the *stop* sign, fewer misled participants were accurate than participants who had received consistent information (41 and 71%, respectively). This study showed that new, incorrect information about a previously experienced event can change the way people remember that event. This “misinformation” effect is now one of the most influential findings in psychology (see Zaragoza, Belli, & Payment, 2006). Numerous studies have replicated and extended these findings, varying from misinformation that alters aspects of the original memory (e.g., remembering a hammer as a screwdriver or remembering a non-existing barn along a country road; Loftus, 1975; McCloskey & Zaragoza, 1985) to the creation of entire events (e.g., being lost in a mall; e.g., Loftus & Pickrell, 1995). Studies have even shown that implausible events may be “remembered” after exposure to misleading information (e.g., witnessing demonic possession, being abducted by an UFO; Mazzoni, Loftus, & Kirsch, 2001; Otgaar, Candel, Merckelbach, & Wade, 2009).

A reviewed subset of studies suggests that, on average, about 31% of participants create memories that incorporate the misinformation (Lindsay, Hagen, Read, Wade, & Garry, 2004). Hyman and Loftus (1998) described three processes that are critically involved in the creation of such false memories. First, the new information needs to be perceived as plausible, which can be achieved by simple interventions. For instance, Mazzoni et al. (2001) showed that merely reading mini-articles about the high frequency of an implausible event (like witnessing demonic possession) increased participants’ ratings of plausibility and likelihood that they had experienced this event. Second, the new information should be visualized. Vivid images with great sensory and perceptual details are more prone to be (falsely) labelled as memories for actual events (Drivdahl & Zaragoza, 2001; Thomas, Bulevich, & Loftus, 2003). Third, a source memory error should occur. This concerns the attribution of the memory's origin to an incorrect source (e.g., to a personal experience, rather than other people, television, or a newspaper; Johnson, Hashtroudi, & Lindsay, 1993).

Only a few studies so far have tested the misinformation effect outside of the laboratory, but these focused on short-term effects. For example, Crombag, Wagenaar, and van Koppen (1996) asked participants about details of a tragedy that took place on October 4, 1992, when an airplane crashed in an apartment building in Amsterdam. The crash received a lot of media coverage in the Netherlands, which included reports of eyewitnesses and videos of the aftermath. Although no video material of the actual crash existed, more than half of the participants said they had seen the video of this moment and a substantial part even “remembered” details about how the plane hit the building and what happened next. Crombag et al. (1996) suggested that dramatic events may be more vulnerable for the misinformation effect than ordinary events, because of their ability to evoke vivid images that interfere with source monitoring.

A recent study by Zhu et al. (2012) tested whether misinformation effects may persist for long periods of time. They showed participants a series of pictures of events (e.g., a picture of a man who puts his wallet in his jacket's outside pocket as part of the series about a girl whose wallet is being stolen). The pictures were followed by narratives that described the events of the pictures; some included accurate information, others included misinformation (e.g., the man put his wallet in his *pants’ pocket*). In a recognition task, questions were asked about the event in the picture (e.g., where did the man put his wallet?). Results indicated that the misinformation provided in the narrative was incorporated in the memory for the picture. About 1.5 years later, participants were retested and some still showed the misinformation effect. However, this was a laboratory study, and it remains unknown whether the effect persists in the long run outside of the laboratory.

Another issue that remains unclear is which individual characteristics increase susceptibility to the misinformation effect. A likely candidate is arousal at time of encoding of the misinformation, because it is enhances memory storage (Cahill & McGaugh, 1998). Therefore, heightened arousal may increase the likelihood that new information is stored and later remembered. With respect to more stable individual differences, studies have found that cognitive ability is negatively correlated with proneness to incorporate misinformation into existing memories (Gudjonsson, 1983; Singh & Gudjonsson, 1992; Zhu et al., 2010a), although other studies have failed to find this correlation (Powers, Andriks, & Loftus, 1979; Salthouse & Siedlecki, 2007). There is also evidence that neuroticism is positively correlated with susceptibility to the misinformation effect (Gudjonsson, 1983; Liebman et al., 2002). However, a recent study showed that harm avoidance, which is associated with neuroticism, correlated negatively with susceptibility to the misinformation effect (Zhu et al., 2010b). To examine the independent predictive value of these variables, they should be included in one study.

The aim of the current study was to test the long-term effect of subtle misinformation outside of the laboratory, and to explore several potential predictors. Since the misinformation effect depends on plausibility, we decided to examine this issue in a convenience sample of Dutch soldiers who had been deployed to Afghanistan, and had been exposed to similar events there. The misinformation related to a fictional deployment-related stressor. Participants were tested in a larger prospective project about vulnerability and resilience factors in risk for PTSD. They were tested about 2 months before deployment (pre-test), and, again about 2 months after returning home (post-test). At the post-test, they received an interview that included questions about exposure to stressors on deployment. At the end, they were given misinformation about an event that had not occurred during their deployment, but which seemed plausible. It involved a brief description about a (harmless) missile attack on their base on New Year's Eve. Participants were merely asked whether they had been exposed to such an event during their deployment. About 7 months later (follow-up), participants were asked to complete a questionnaire with items that referred to deployment-related stressors. For each stressor, participants indicated whether they had or had not experienced it during their deployment. One item referred to a missile attack on New Year's Eve. Potential predictors of the misinformation effect were arousal, cognitive ability, and neuroticism. It may be speculated that soldiers with more stressors on deployment were better able to construct vivid images related to the misinformation and found it more plausible. Therefore, the interaction between stressors on deployment and arousal was also included as a potential predictor.

It was predicted that a substantial minority of the participants would show the misinformation effect at the follow-up (i.e., they would report having experienced the missile attack on New Year's evening). It was also predicted that arousal, the number of deployment-related stressors, the interaction between arousal and stressors, cognitive ability, and neuroticism would be associated with the misinformation effect.

## Method

### Participants and procedure

Participants included 249 Dutch Royal Army soldiers (98% male, mean age = 23.8 years, *SD*=4.9), who enrolled in a prospective study about PTSD before their 4-month deployment to Afghanistan in 2010 (see Lommen, Engelhard, Sijbrandij, van den Hout, & Hermans, 2013). For most participants the highest attained educational level was secondary school (92%), but for some it was primary school (2%) or college/university (6%). About 34% of participants of this sample were married or cohabiting, 38% were in a relationship but not cohabiting, and 28% were single. About 43% had not been deployed before.

At the pre-test, participants completed the questionnaire about neuroticism. At the post-test, 247 participants (99%) were retested, and received an interview that included assessment of deployment-related stressors. After that, participants were given subtle misinformation about a missile attack at the base on New Year's Eve. Questionnaires measuring PTSD symptom severity during the past month and exposure to stressors on deployment were also administered. At the follow-up test, 221 participants (89%) completed the deployment stressor questionnaire again, to which an item about the missile attack on New Year's Even was now added. A total of 181 participants also completed the cognitive ability test. Non-response was partly due to participants who were unreachable after a transfer, or withdrew from the study.

For recruitment details see Lommen et al. (2013). Participation was voluntary without financial compensation. Participants provided oral and written informed consent at the pre-test and again at the post-test. The Medical Ethical Committee of Maastricht University approved this study.

### Measures

PTSD symptom severity was assessed with the Dutch version (Engelhard, Arntz, & van den Hout, 2007) of the Posttraumatic Symptom Scale—Self Report (PSS; Foa, Riggs, Dancu, & Rothbaum, 1993). Participants were asked to rate the 17 DSM-IV PTSD symptoms on a 0 (*not at all*) to 3 (*almost always*) scale for the prior month, with respect to the deployment-related event(s) that troubled them the most. The sum score of the hyperarousal subscale (PSS-H; 5 items) was used. Cronbach's alpha at the post-test was .81 for the total scale, and .54, .72, and .68 for the subscales (re-experiencing, avoidance, and hyperarousal, respectively). The PSS is a valid and reliable measure (Engelhard et al., 2007; Foa et al., 1993).

Stressful events were assessed with the Dutch version (Engelhard & van den Hout, 2007) of the Potentially Traumatizing Events Scale (PTES; Maguen, Litz, Wang, & Cook, 2004). To adjust the scale to deployment to Afghanistan, one of the original 21 items that represent war-zone related stressors, was omitted (“patrolling areas where there were land mines”), and four were added (“Having injured civilians due to own action” and “being formally told that a colleague got killed”; Engelhard & van den Hout, 2007, “seeing dead or injured Afghan soldiers or police”, and “conflict situation with Afghan police”). Another item was adjusted to the situation in Afghanistan (“patrolling through the zone of separation” was changed to “stand guard during patrol”). For each of the 24 stressors, participants indicated whether or not they had experienced it on their deployment to Afghanistan. The number of endorsed stressors was used (range 0–24).

Cognitive ability was assessed with the Standard Progressive Matrices (Raven, 1976), which is an abstract reasoning task that consists of 60 multiple-choice items. The number of correct answers was computed (range 0–60).

Neuroticism was assessed with the Dutch version (Sanderman, Arrindell, Ranchor, Eysenck, & Eysenck, 1991) of the neuroticism scale of the Eysenck Personality Questionnaire—short version (EPQ-N; Eysenck, & Eysenck, 1975). This widely-used scale consists of 22 items that can be answered with *yes* (= 1) or *no* (= 0). The sum score was used. Psychometric properties of this scale are good (Sanderman et al., 1991).

### Misinformation effect

At the post-test, participants were provided new information about an event that did not take place during their deployment, that is, a (harmless) missile attack at the base on New Year's Eve. We provided a short description of the event including some sensory details (e.g., sound of explosion, sight of gravel after the explosion). After that, participants were asked if they had experienced a missile attack on New Year's Eve during their deployment to Afghanistan. At follow-up, “Missile attack on New Year's Eve” was added to the PTES. The misinformation effect was considered as present if participants indicated they had experienced the missile attack on New Year's Eve.

### Statistical analysis

The Raven score was not normally distributed. Therefore, the scores of four outliers were replaced by *M*-2.5×*SD* to obtain a normal distribution. Furthermore, eight participants reported that they had experienced the fictional event at the post-test and were excluded from the analyses. To explore factors that may contribute to the misinformation effect susceptibility, Pearson correlations were computed between arousal (PSS-H), stressors (PTES), cognitive ability (Raven), neuroticism (EPQ-N) and the misinformation effect. Then a logistic regression analysis was run, with the misinformation effect as dependent variable. The PSS-H×PTES interaction and those variables that correlated significantly with the misinformation effect were centered and included as independent variables.

## Results

### Non-response analysis

There were no differences regarding scores on EPQ-N at pre-test, PTES at post-test, or PSS-H at post-test between participants who completed the Raven, largest *t*=1.41, *p*=.16, and those who have not, or between responders and non-responders at follow-up, largest *t*=.76, *p*=.45, which suggested an absence of selection bias.

### Misinformation effect

A total of 213 participants received the misinformation at the post-test and indicated they had not experienced this event during their deployment. Seven months later, 55 (26%) reported that they had experienced this event on their deployment. More soldiers who had not been deployed before showed the misinformation effect (*n=*30), compared to soldiers who had been deployed before (*n*= 25), *χ* ^2^=4.30, *df=*1, *p*=.04.

### Susceptibility to the misinformation effect

Correlations (Table 1) showed that the misinformation effect was positively associated with the PTES and negatively associated with the Raven, but not associated with the PSS-H and EPQ-N. The multiple logistic regression analysis showed that the PSS-H×PTES interaction^1^ and Raven were significant predictors of the misinformation effect, but prior deployment was not (*p*>.05). The model including only the significant predictors (Table 2) showed good fit according to the Hosmer and Lemeshow Goodness-of-fit test, *χ* ^2^=2.79, *df=*8, *p*=.95. According to the Box-Tidwell approach, the assumption of linearity of logits was not violated, smallest *p*=.11. The explained variance by the tested model ranged from 12% (Cox & Snell *R* ^2^ and McFadden *R* ^2^) to 18% (Nagelkerke *R* ^2^).

**Table 1:** Descriptive statistics and Pearson correlations

	1	2	3	4	*M* (SD)		Range
1. Misinformation effect					No (*n*=158)	Yes (*n*=55)	
2. PSS-H	.09				1.68 (2.03)	2.11 (2.18)	0–11
3. PTES	.31*	.21*			13.30 (4.43)	16.64 (4.85)	0–24
4. Raven	−.26*	.02	−.18*		50.07 (5.28)	46.62 (6.22)	33–60
5. EPQ-N	.02	.39*	.06	−.02	3.29 (3.42)	3.44 (3.73)	0–16

*Note*. PSS-H=Posttraumatic Symptom Scale—hyperarousal subscale; PTES=Potentially Traumatizing Events Scale; Raven=Standard Progressive Matrices; EPQ-N=Eysenck Personality Questionnaire—neuroticism scale. The number of participants was smaller for the variable Raven (*no*=134, *yes*=42).

*p*<.05.

**Table 2:** Logistic regression analyses predicting the misinformation effect

Model	*B* (SE)	Wald	*p*	OR (95% CI)
PSS-H	−0.07 (.10)	0.49	.49	0.93 (0.76–1.14)
PTES	0.12 (.05)	7.05	.01	1.13 (1.03–1.24)
PSS-H×PTES interaction	0.06 (.03)	4.64	.03	1.06 (1.01–1.12)
Raven	−0.10 (.03)	8.71	<.01	0.91 (0.85–0.97)

*Note*. PSS-H=Posttraumatic Symptom Scale—hyperarousal subscale; PTES=Potentially Traumatizing Events Scale; Raven=Standard Progressive Matrices; EPQ-N=Eysenck Personality Questionnaire—neuroticism scale.

With respect to the Raven, with each point of increase on the Raven test, the logit of the misinformation effect decreased with .10. In terms of odds, the chance on the misinformation effect decreased with a factor of 0.91 (9%) with each point of increase on the Raven.

**Fig. 1:**
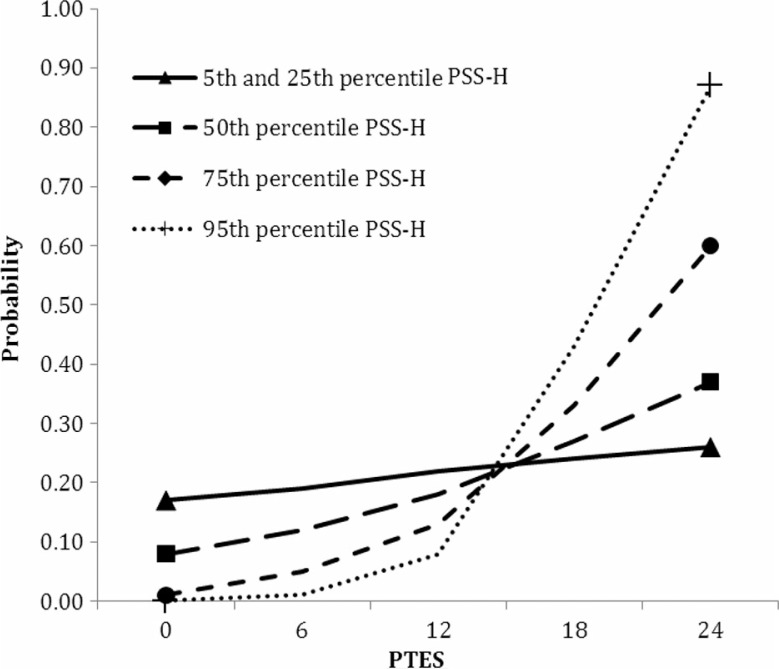
Plotted PSS-H×PTES interaction, with separate lines representing the percentiles of PSS-H, and the probability of the misinformation effect at the Y-axis.

Figure 1 shows the PSS-H×PTES interaction effect, with separate lines for the PSS-H percentiles 5, 25, 50, 75, and 95 and the mean Raven score. Percentile 5 represents the 5% with the lowest scores on the PSS-H; percentile 95 represents the 5% highest scores on the PSS-H. The figure shows that the probability of the misinformation effect for low PSS-H scores was hardly affected by PTES scores. In contrast, the probability of the misinformation effect in high PSS-H scores depended on PTES scores, with higher scores on both variables resulting in the highest probability scores. In other words, probability of the misinformation effect increased with the number of stressors when arousal was high, but the number of stressors did hardly influence the probability of the misinformation effect when arousal was low.

## Discussion

The main findings can be summarized as follows. First, as hypothesized, a substantial minority of soldiers (26%) reported that they had experienced the fictional missile attack on New Year's Eve during their deployment, about 7 months after they received information about this event. None of them had indicated 2 months after deployment that they had experienced this event. This finding is in line with the mean percentage of participants showing the misinformation effect in a reviewed subset of studies (31%; Lindsay et al., 2004). The current study extends these prior findings to a longer period (i.e., about 7 months). Second, susceptibility to the misinformation effect was related to the interaction between arousal and the number of stressors on deployment, and to lower cognitive ability. Susceptibility was relatively low when arousal was high and the number of deployment-related stressors was low, but susceptibility increased when both arousal and the number of stressors were high. How can this interaction be explained? Arousal at time of encoding enhances memory (e.g., Cahill & McGaugh, 1998), and the number of experienced stressors may influence the ease with which related misinformation evokes vivid images, and may affect the perceived plausibility. Therefore, new information may not be remembered if arousal at the time of encoding is low, independently of the plausibility and visual processing of the information. However, when arousal is high, the misinformation may be remembered, particularly if it is plausible and evokes vivid images. Results concerning the association between lower cognitive ability and susceptibility to the misinformation effect replicate earlier findings (Gudjonsson, 1983; Singh & Gudjonsson, 1992; Zhu et al., 2010a). It seems plausible that cognitive ability is associated with memory for details (e.g., including the source of the information). If information is less accurately stored, it may be more susceptible for misinformation because the correct information cannot be remembered. Cognitive ability scores of the current sample were lying within the average range (Raven, Court, & Raven, 1992), which suggests that relatively small differences in cognitive ability affect susceptibility for the misinformation effect. Similar results have been shown in studies on suggestibility in children, although the association ceased to exist in above-average levels of cognitive ability (Gignac & Powell, 2006). Future studies may investigate the relation in a range of cognitive ability levels and may elucidate the underlying process.

Several limitations of the current study should be considered before definite conclusions can be made. First, the main limitation is that the misinformation effect was based on a single item on a questionnaire. It remains unclear whether participants really remembered the event, or perhaps misunderstood the question. They may also have confused the event with an actual attack during prior deployments, although this is unlikely because participants without prior deployments were more prone to the misinformation effect, and they did not report the event at the post-test. More detailed questions may address this issue in future studies. Second, because we did not include a control group that did not receive misinformation, we cannot exclude that changes in report of the fictional event may reflect “general” increased recall over time (e.g., Engelhard, van den Hout, & McNally, 2008; King et al., 2000; Southwick, Morgan, Nicolaou, & Charney, 1997). However, increased recall for other stressors ranged from 2 to 19% with an average of 10%, so it seems unlikely that our results could be explained by a general increase in recall. Third, other potential predictors of the misinformation effect, such as individual differences in vividness of mental imagery (e.g., fantasy proneness and hypnotisability; Barnier & McConkey, 1992; Hyman & Billings, 1998; Jelicic et al., 2006) were not included. Fourth, the arousal measure related to arousal over the past month, and arousal at time of encoding the misinformation was not included. Nonetheless, one might expect a substantial correlation between arousal over the past month and state arousal, as has been found for state and trait anxiety (e.g., Spielberger & Sydeman, 1994). Fifth, the Cronbach's alpha of the PSS subscales was rather low, since values of .70–.95 are considered as acceptable. This may be due to the small number of items in this subscale. Sixth, behavioral effects of the misinformation were not assessed. It seems unlikely that the current mild manipulation resulted in behavioral effects. However, there is evidence that actual behavior may be affected by stronger manipulation, that is, when participants are convinced that the fictional event happened to them. One study (Geraerts et al., 2008) found that participants who believed the false suggestion that they had gotten ill as a child after eating egg salad, avoided subsequent consumption of egg salad.

Despite these limitations, the findings suggest that the misinformation effect may have long-term effects outside of the laboratory. With respect to clinical implications, this study may add to the awareness about the malleability of memory. In line with the body of literature on memory malleability, it is important for clinicians to understand that memory for a potentially traumatic event is not immutable (see also Engelhard, van den Hout, & McNally, 2008). New information, from whatever source, can be incorporated into existing memories and can change the way people remember events. Especially individuals with lower cognitive ability, high arousal at the time of encoding the information and more related experiences may be prone to the misinformation effect.

## Footnotes

1 To rule out that the interaction effect was driven by PTSD symptoms instead of arousal, the analysis was reran with the other PTSD symptom clusters (re-experiencing and avoidance), which revealed non-significant interactions.

## References

[CIT0001] Barnier A. J, McConkey K. M (1992). Reports of real and false memories: The relevance of hypnosis, hypnotizability, and context of memory test. Journal of Abnormal Psychology.

[CIT0002] Cahill L, McGaugh J. L (1998). Mechanisms of emotional arousal and lasting declarative memory. Trends in Neurosciences.

[CIT0003] Crombag H. F. M, Wagenaar W. A, van Koppen P. J (1996). Crashing memories and the problem of “source monitoring”. Applied Cognitive Psychology.

[CIT0004] Drivdahl S. B, Zaragoza M. S (2001). The role of perceptual elaboration and individual differences in the creation of false memories for suggested events. Applied Cognitive Psychology.

[CIT0005] Engelhard I. M, Arntz A, van den Hout M. A (2007). Low specificity of symptoms on the post-traumatic stress disorder (PTSD) symptom scale: A comparison of individuals with PTSD, individuals with other anxiety disorders, and individuals without psychopathology. British Journal of Clinical Psychology.

[CIT0006] Engelhard I. M, van den Hout M. A (2007). Preexisting neuroticism, subjective stressor severity, and posttraumatic stress in soldiers deployed to Iraq. Canadian Journal of Psychiatry.

[CIT0007] Engelhard I. M, van den Hout M. A, McNally R. J (2008). Memory inconsistency for traumatic events in Dutch soldiers deployed to Iraq. Memory.

[CIT0008] Eysenck H. J, Eysenck S. B. G (1975). Manual of the Eysenck Personality Questionnaire.

[CIT0009] Foa E. B, Riggs D. S, Dancu C. V, Rothbaum B. O (1993). Reliability and validity of a brief instrument for assessing post-traumatic stress disorder. Journal of Traumatic Stress.

[CIT0010] Geraerts E, Bernstein D.M, Merckelbach H, Linders C, Raymaekers L, Loftus E.F (2008). Lasting false beliefs and their behavioral consequences. Psychological Science.

[CIT0011] Gignac G. E, Powell M. B (2006). A direct examination of the nonlinear (quadratic) association between intelligence and suggestibility in children. Applied Cognitive Psychology.

[CIT0012] Gudjonsson G. H (1983). Suggestibility, intelligence, memory recall and personality: An experimental study. The British Journal of Psychiatry.

[CIT0013] Hyman I. E, Billings F. J (1998). Individual differences and the creation of false childhood memories. Memory.

[CIT0014] Hyman I. E, Loftus E. F (1998). Errors in autobiographical memory. Clinical Psychology Review.

[CIT0015] Jelicic M, Smeets T, Peters M. J. V, Candel I, Horselenberg R, Merckelbach H (2006). Assassination of a controversial politician: Remembering details from another non-existent film. Applied Cognitive Psychology.

[CIT0016] Johnson M. K, Hashtroudi S, Lindsay D. S (1993). Source monitoring. Psychological Bulletin.

[CIT0017] King D. W, King L. A, Erickson D. J, Huang M. T, Sharkansky E. J, Wolfe J (2000). Posttraumatic stress disorder and retrospectively reported stressor exposure: A longitudinal prediction model. Journal of Abnormal Psychology.

[CIT0018] Liebman J. I, McKinley-Pace M. J, Leonard A. M, Sheesley L. A, Gallant C. L, Renkey M. E (2002). Cognitive and psychosocial correlates of adults’ eyewitness accuracy and suggestibility. Personality and Individual Differences.

[CIT0019] Lindsay D. S, Hagen L, Read J. D, Wade K. A, Garry M (2004). True photographs and false memories. Psychological Science.

[CIT0020] Loftus E. F (1975). Leading questions and the eyewitness report. Cognitive Psychology.

[CIT0021] Loftus E. F, Miller D. G, Burns H. J (1978). Semantic integration of verbal information into a visual memory. Journal of Experimental Psychology: Human Learning and Memory.

[CIT0022] Loftus E. F, Palmer J. C (1974). Reconstruction of automobile destruction: An example of the interaction between language and memory. Journal of Verbal Learning and Verbal Behavior.

[CIT0023] Loftus E. F, Pickrell J. E (1995). The formation of false memories. Psychiatric Annals.

[CIT0024] Lommen M. J. J, Engelhard I. M, Sijbrandij E. M, van den Hout M. A, Hermans D (2013). Pre-trauma individual differences in extinction learning predict posttraumatic stress. Behaviour Research and Therapy.

[CIT0025] Maguen S, Litz B. T, Wang J. L, Cook M (2004). The stressors and demands of peacekeeping in Kosovo: Predictors of mental health response. Military Medicine.

[CIT0026] Mazzoni G. A. L, Loftus E. F, Kirsch I (2001). Changing beliefs about implausible autobiographical events: A little plausibility goes a long way. Journal of Experimental Psychology: Applied.

[CIT0027] McCloskey M, Zaragoza M (1985). Misleading postevent information and memory for events: Arguments and evidence against memory impairment hypotheses. Journal of Experimental Psychology: General.

[CIT0028] Otgaar H, Candel I, Merckelback H, Wade K. A (2009). Abducted by a UFO: Prevalence information affects young children's false memories for an implausible event. Applied Cognitive Psychology.

[CIT0029] Powers P. A, Andriks J. L, Loftus E. F (1979). Eyewitness accounts of females and males. Journal of Applied Psychology.

[CIT0030] Raven J. C (1976). Standard progressive matrices.

[CIT0031] Raven J. C, Court J. H, Raven J (1992). Manual for Raven's progressive matrices and vocabulary scales.

[CIT0032] Salthouse T. A, Siedlecki K. L (2007). An individual difference analysis of false recognition. The American Journal of Psychology.

[CIT0033] Sanderman R, Arrindell W. A, Ranchor A. V, Eysenck H. J, Eysenck S. B. G (1991). Eysenck Personality Questionnaire (EPQ).

[CIT0034] Singh K. K, Gudjonsson G. H (1992). Interrogative suggestibility among adolescent boys and its relationship with intelligence, memory, and cognitive set. Journal of Adolescence.

[CIT0035] Spielberger C. D, Sydeman S. J, Maruish M. E (1994). State-trait anxiety inventory and state-trait anger expression inventory. The use of psychological testing for treatment planning and outcome assessment.

[CIT0036] Southwick S. M, Morgan C. A, Nicolaou A. L, Charney D. S (1997). Consistency of memory for combat-related traumatic events in veterans of operation Desert Storm. American Journal of Psychiatry.

[CIT0037] Thomas A. K, Bulevich J. B, Loftus E. F (2003). Exploring the role of repetition and sensory elaboration in the imagination inflation effect. Memory & Cognition.

[CIT0038] Zaragoza M. S, Belli R. F, Payment K. E, Gary M, Hayne H (2006). Misinformation effects and the suggestibility of eyewitness memory. Do justice and let the sky fall: Elizabeth F. Loftus and her contributions to science, law, and academic freedom.

[CIT0039] Zhu B, Chen C, Loftus E. F, He Q, Chen C, Lei X (2012). Brief exposure to misinformation can lead to long-term false memories. Applied Cognitive Psychology.

[CIT0040] Zhu B, Chen C, Loftus E. F, Lin C, He Q, Chen C (2010a). Individual differences in false memory from misinformation: Cognitive factors. Memory.

[CIT0041] Zhu B, Chen C, Loftus E. F, Lin C, He Q, Chen C (2010b). Individual differences in false memory from misinformation: Personality characteristics and their interactions with cognitive ability. Personality and Individual Differences.

